# Coordination between *GROWTH-REGULATING FACTOR1* and *GRF-INTERACTING FACTOR1* plays a key role in regulating leaf growth in rice

**DOI:** 10.1186/s12870-020-02417-0

**Published:** 2020-05-08

**Authors:** Yuzhu Lu, Yunlong Meng, Jia Zeng, Ying Luo, Zhen Feng, Liying Bian, Suyun Gao

**Affiliations:** 1 Jiangsu Key Laboratory of Crop Genetics and Physiology/ Key Laboratory of Plant Functional Genomics of the Ministry of Education, Yangzhou University, Yangzhou, 225009 China; 2 Joint International Research Laboratory of Agriculture and Agri-Product Safety, the Ministry of Education of China, Yangzhou University, Yangzhou, 225009 Jiangsu China; 3 College of Bioscience and Biotechnology, Yangzhou University, Yangzhou, 225009 China

**Keywords:** *OsGRF1*, *OsGIF1*, miR396, Leaf growth, Stress response

## Abstract

**Background:**

The interactions between Growth-regulating factors (GRFs) and GRF-Interacting Factors (GIFs) have been well demonstrated but it remains unclear whether different combinations of GRF and GIF play distinctive roles in the pathway downstream of the complex.

**Results:**

Here we showed that *OsGRF1* and *OsGIF1* synergistically regulate leaf growth in rice. The expression of *OsGIF1* emerged in all tissues with much higher level while that of *OsGRF1* appeared preferentially only in the stem tips containing shoot apical meristem (SAM) and younger leaves containing leaf primordium. Overexpression of an OsmiR396-resistant version of *mOsGRF1* resulted in expanded leaves due to increased cell proliferation while knockdown of *OsGRF1* displayed an opposite phenotype. Overexpression of *OsGIF1* did not exhibit new phenotype while knockdown lines displayed pleiotropic growth defects including shrunken leaves. The crossed lines of *mOsGRF1* overexpression and *OsGIF1* knockdown still exhibited shrunk leaves, indicating that *OsGIF1* is indispensable in leaf growth regulated by *OsGRF1*. The expression of *OsGRF1* could be upregulated by gibberellins (GAs) and downregulated by various stresses while that of *OsGIF1* could not.

**Conclusion:**

Our results suggest that *OsGIF1* is in an excessive expression in various tissues and play roles in various aspects of growth while *OsGRF1* may specifically involve in leaf growth through titrating *OsGIF1.* Both internal and external conditions impacting leaf growth are likely via way of regulating the expression of *OsGRF1*.

## Background

Interactions between transcription factors (TFs) and their coactivators are usually essential in regulating downstream genes expression and thus in properly modulating individual growth. Despite a wide range of genes’ interactions existed in vivo, only limited numbers have been identified due to the lack of assuredly reliable methods. In plants, GROWTH-REGULATING FACTOR (GRF) and GRF-INTERACTING FACTOR (GIF) were well known to interact with each other and this complex duo has been proved to participate in many aspects of the development and growth in plants [15, 17, 21, 22, 31].

*OsGRF1* is the first member found to be induced by gibberellic acid (GA) in rice [43, 44]. Based on the features of OsGRF1’s amino acid sequence, a family of 12 members has been found in rice [6]. This plant-specific family was defined by two conserved domains, QLQ (Gln, Leu, Gln) and WRC (Trp, Arg, Cys), in the N-terminal region of GRF proteins. The QLQ domain is essential for protein-protein interaction [17] and WRC domain comprising a C_3_H motif is believed to bind DNA with its nuclear localization signal (NLS) [6]. The roles of GRFs were initially thought to regulate the growth of leaf and stem [13, 16, 18, 44]. Thereafter growing number of studies reported other functions of GRFs, such as seed and root development, stress response, flowing, and plant longevity [3, 7, 11, 19, 24, 26, 33]. As a highly conserved family, GRFs have been found in all land plants including *Arabidopsis thaliana*, *Brassica napus*, *Glycine max*, *Solanum tuberosum*, *Zea mays*, the moss *Physcomitrella patens* [2, 8, 19, 20, 27, 32, 46, 51, 53]. Most members of *GRFs* are negatively regulated by miR396, which cleave their targets at the transcript level [14]. In rice, 11 of 12 members of *OsGRFs* are targets of OsmiR396, except *OsGRF11* [40].

MiR396 was firstly identified in *Arabidopsis* and rice by computational and experimental means [14, 40, 49, 50]. Like GRFs, miR396 family is also a highly conserved plant microRNA family found in all land plants [1]. MiR396 has been demonstrated to be involved in various aspects of plant growth and development [4, 5, 9, 11, 25, 26, 29, 35–37, 47]. As a regulatory molecule, the roles of miR396 depend on the functions of its targets as well as the ways how it regulates its targets.

Compared with *GRF* family which usually comprises 8–20 numbers, *GIF* family is much smaller with only few members, usually below 5 copies, in different plants [31]. However, the phenomenon that *GIF* genes exist in most eukaryotic species including embryophytes, green algae, and metazoan shows this family is more conserved than *GRF* family [17]. The amino acid sequences of GIF are featured by having two domains, SNH (SYT N-terminal homology) and QG, which is rich of glutamine (Q) and glycine (G). Binding and Y2H assays demonstrated that the GRF QLQ domain and GIF SNH domain mediate the interaction between the two families [13, 17, 26]. In *Arabidopsis*, the interactions between different AtGRFs and AtGIFs members have been well identified [7, 24, 45]. So far, it still remains unclear whether different combinations of GRF and GIF play their unique roles in the downstream of the complex. Interestingly, overexpression of ZmGRF10, which has no transactivation activity due to the lack of almost entire C-terminal domain, was found to fine-tune the homeostasis of the GRF-GIF complex via way of competitive combination [48]. Also, different combinations of GRFs and GIFs have been observed in the different regions of maize leaf [30]. These results showed that the combinations between different members of GRFs and GIFs are widely existed and in a competitive way.

Here we probed into the precise titration relationship between *OsGRF1* and *OsGIF1*. By analyzing their expression and the phenotypes of the transgenic lines, we proposed a coordinated relationship between them.

## Results

### Different expression patterns of *OsGIF1* and *OsGRF1*

**Fig. 1:**
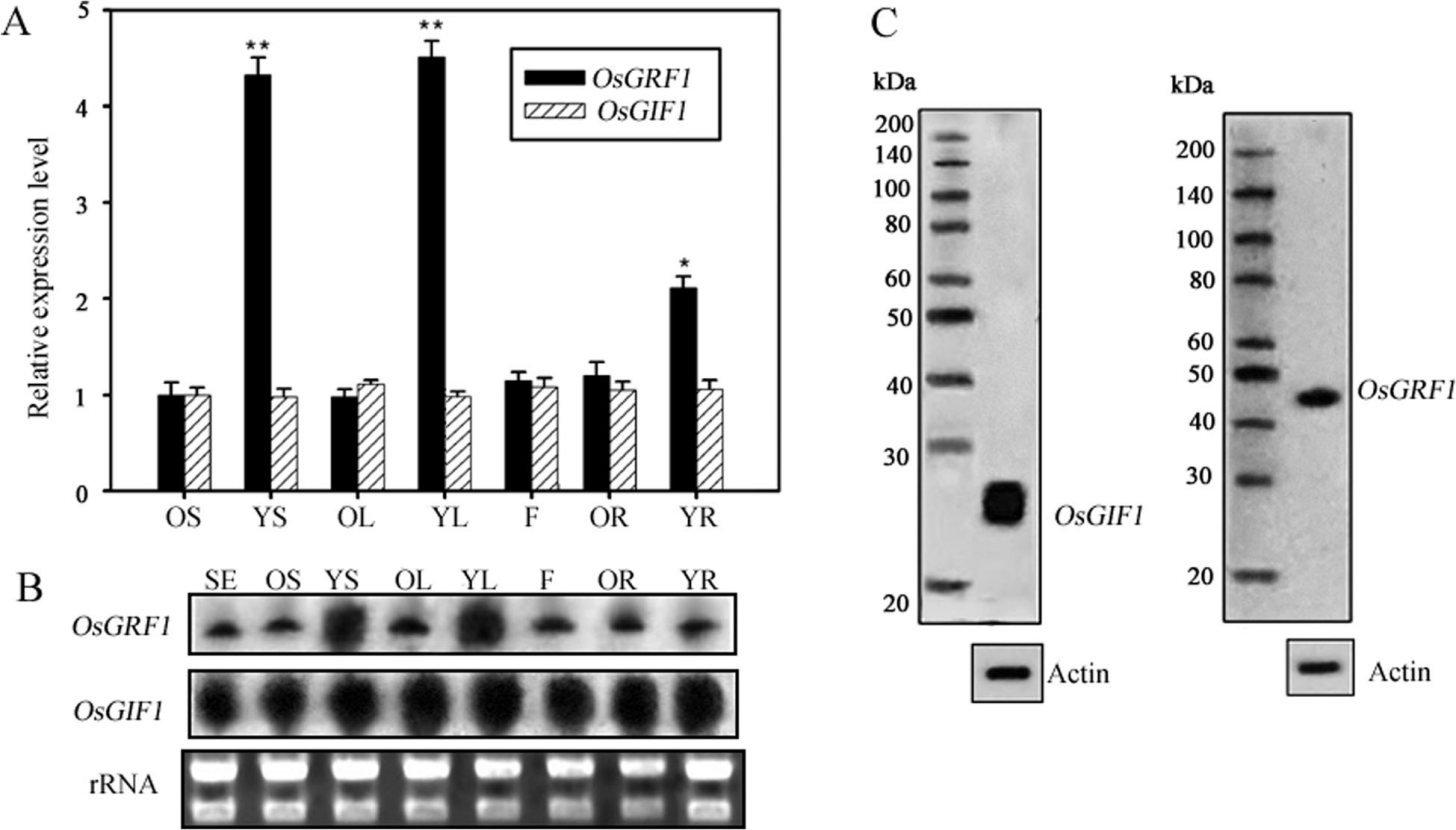
The expression patterns of *OsGRF1* and *OsGIF1* in rice. **a** The expression levels of *OsGRF1* and *OsGIF1* in flowers (adult plants) and different tissues of the 4-week-old seedlings in non-transformed rice (NT). Expression was analyzed by qRT-PCR. *, Significant difference at *P* < 0.05, **, Significant difference at *P* < 0.01 compared with expression in Older Stems by Student’s t-test (*n* = 3; means ± SDs). OS: Older Stem, basal internodes in stem of 4-week-old-seedlings; YS: Younger Stem, 5-mm-long shoot tips containing SAM (Shoot Apical Meristem) of 4-week-old-seedlings; OL: Older Leaf, the leaves in basal shoot of 4-week-old seedlings; YL: Younger Leaf, leaf tips and leaf primordium of 4-week-old-seedlings; F: Flowers in adult stage; OR: Older Root, the basal region in roots of 4-week-old seedlings; YR: Younger Root, 5-mm-long root tips of 4-week-old seedlings. **b** The RNA levels of *OsGRF1* and *OsGIF1* analyzed by northern blot in different tissues of the non-transformed rice (NT). Total RNA from 1-week-old-seedlings (lane SE) and different tissues as described in (A), including older stem (lane OS), Younger stem containing SAM (Shoot Apical Meristem) (lane YS), older leaf (lane OL), younger leaf (lane YL), the flowers in adult stage (lane F), older root (lane OR), and younger root (lane YR) was loaded and electrophoresed. Then the electrophoretic products were transferred and probed by labeled anti-sense sequences. The rRNA bands were visualized by ethidium bromide staining and served as loading control. **c** The protein levels of OsGIF1 and OsGRF1 analyzed by western blot. Total protein extracted from 2-week-old seedlings of the non-transformed plants (NT) was immunoblotted by anti-OsGIF1 (the left) and anti-OsGRF1 (the right) respectively. Actin immunoblotted by anti-Actin was served as control

Although the interactions between different GRFs and GIFs have been well tested ([13, 17, 21]; Lee et al.,2014 [26];), the precise functions of different combinations still remain unclear. There are 12 members of *OsGRF* and 2 members of *OsGIF* in rice (*Oryza sativa ssp. japonica,* [6, 34]). Earlier studies revealed that *OsGRF1* is a GA induced gene and can affect the stem elongation in Arabidopsis [6, 43, 44]. The functions of *OsGIF1* have been reported to be involved in regulating growth of multiple organs such as leaves, stems and grains [10, 23]. Investigating genes expression patterns is necessary for probing into their functions because genes expression patterns are usually consistent with their roles. Here, we chose *OsGRF1* and *OsGIF1* as objectives to fully investigate their expression profiles. We selected flowers from the adult plants and different older and younger tissues such as leaves, stems, and roots from 4-week-old seedlings as objects for analyzing genes expression. Quantitative Reverse Trancription-PCR (qRT-PCR) showed that the expression levels of *OsGRF1* were relatively higher in the younger tissues including younger leaves, shoots, and roots, especially in shoot apical meristem (SAM) and leaf primordium (Fig. 1a). By contrast, the expression of *OsGIF1* seemed to be constitutive with similar levels in almost all tested tissues (Fig. 1a).

qRT-PCR may show the expressional tendencies of *OsGRF1* and *OsGIF1* in different tissues, but cannot reflect the intensity differences of the expressions. To further compare their expression level, especially for the intensities between *OsGRF1* and *OsGIF1*, we measured the two genes’ expression by northern blot. We elaborately employed two probes containing same content of radioactively labeled α-^32^P-dCTP which was inserted into probes by PCR for hybridizing the two genes respectively. As Fig. 1b shown, the RNA abundance of *OsGIF1* was much higher than that of *OsGRF1* in all tested tissues, even in younger leaves and shoots where the expression levels *OsGRF1* were also relatively higher. Overall, the expression levels detected by northern blot and qRT-PCR were consistent with each other (Fig. 1a and b). These results indicated that the expression of *OsGIF1* is in a constitutive manner with much higher level, however, the expression of *OsGRF1* displays a tissue-specific preference with relatively lower level. To further investigate the two genes expression on protein level, total protein was extracted from 2-week-old seedlings and was immunoblotted by anti-OsGIF1 and anti-OsGRF1 respectively. The molecular weight of the OsGIF1 was about 25 kDa while that of the OsGRF1 is about 43.5 kDa. As shown in Fig. 1c, the blot intensity of OsGIF1 was much stronger than that of OsGRF1, further indicating the protein abundance OsGIF1 was more abundant than OsGRF1.

### The expression of *OsGRF1* can be regulated by phytohormones and stresses while that of *OsGIF1* cannot

**Fig. 2:**
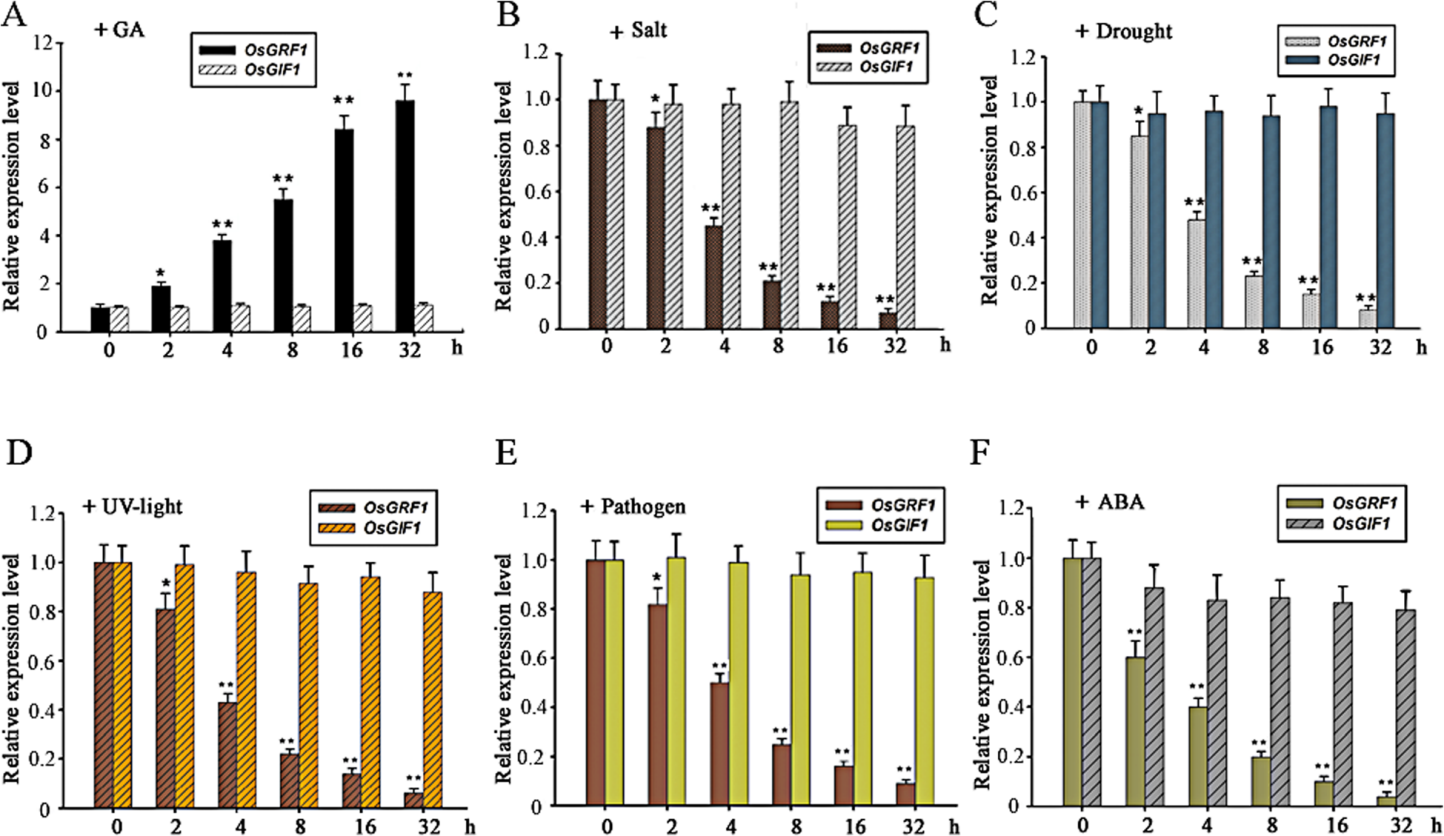
Response of *OsGRF1* and *OsGIF1* to GA, ABA, and stresses. Time course analysis of expressions of *OsGRF1* and *OsGIF1* in response to gibberellins (GA), salt, drought, UV-light, pathogen, and ABA. 2-week-old seedlings were incubated into N6 solution containing 50 μM GA_3_ (**a**) or 200 mM NaCl (**b**) or 1 μM ABA (F) for designed time. 2-week-old seedlings were transplanted into 25% PEG (polyethylene glycol) (**c**), or exposed to 100 μmol m^− 2^ s^− 1^ ultraviolet (**d**), or sprayed with 3 × 10^5^ spore ml^− 1^*Magnaporthe grisea* (**e**) for designated time respectively. Expression was analyzed by qRT-PCR. *, Significant difference at *P* < 0.05, **, Significant difference at *P* < 0.01 compared with No treatment by Student’s t-test (n = 3; means ± SDs)

It is well known that the *OsGRF1* and most other *OsGRFs* are GA-inducible [6, 43, 44]. As a kind of basic plant hormone, gibberellins (GAs) are often in a pivotal hub of different pathways. Usually the concentration of endogenous gibberellins is likely affected by other factors, such as biotic and abiotic stresses [42]. Additionally we did not know whether or how *OsGRF1* and *OsGIF1* respond to these factors. We chose 2-week-old seedlings exposed to different treatments including GA, salt, drought, UV, pathogen, and ABA for designated time. Then the total RNA from these seedlings was extracted and the two genes’ expressions were measured by qRT-PCR respectively. As expected, the expression of *OsGRF1* was gradually increased with the extension of GA_3_ treatment, while that of *OsGIF1* was not affected during the identical period (Fig. 2a). By contrast, the expressions of *OsGRF1* were gradually reduced under the treatments of ABA and various stresses (Fig. 2b to f). Similar as GA treatment, the expressions of *OsGIF1* were also unaffected in the other treatments (Fig. 2). Interestingly, the variation of *OsGRF1* expression under ABA treatment was more remarkable than other stresses, and the expression of Os*GIF1* was also slightly fallen under ABA treatment (Fig. 2b to f), indicating that ABA, one of stresses-associated hormones, may have a rapid effect upon the genes’ expression than other stresses. These results showed that the expression of *OsGRF1* could be regulated by various factors, but that of *OsGIF1* could not.

### Overexpression of miR396-resistant version of *OsGRF1* results in expanded leaves

**Fig. 3:**
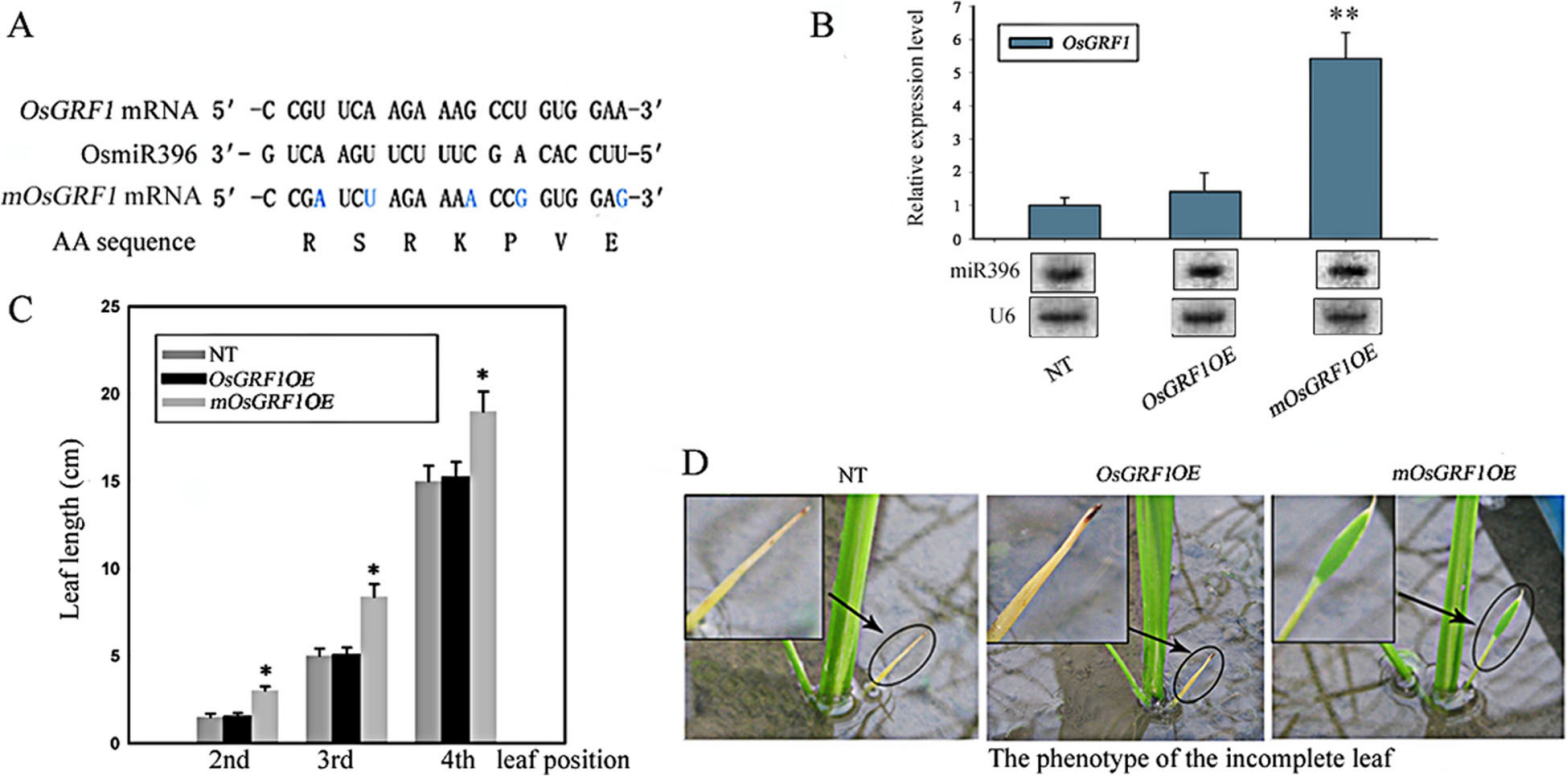
The Phenotypes of the non-transformed rice, *OsGRF1OE*, and *mOsGRF1OE.***a** The regions complementary to OsmiR396 in *OsGRF1* and *mOsGRF1* (mutated *OsGRF1*) mRNA and the corresponding amino acid sequence. The mutated sites (shown in blue) in *mOsGRF1* were artificially brought into to reduce the degree of the complementarity to OsmiR396 without alteration of amino acid sequence. **b** The RNA levels of OsmiR396 and *OsGRF1* in the seedlings of the non-transformed plants (NT), *OsGRF1OE*, and *mOsGRF1OE.* OsmiR396 was detected by northern blot and U6 was served as loading control. *OsGRF1* was analyzed by qRT-PCR, **, Significant difference at *P* < 0.01 compared with expression in the non-transformed plants (NT) by Student’s t-test (n = 3; means ± SDs). **c** The length of different leaves in 4-week-old seedlings of the non-transformed plants (NT), *OsGRF1OE*, and *mOsGRF1OE.* *, Significant difference at *P* < 0.05 compared with the non-transformed plants (NT) by Student’s t-test (*n* = 5; means ± SDs). **d** The morphology of the incomplete leaf in 4-week-old seedlings of the non-transformed plants (NT), *OsGRF1OE*, and *mOsGRF1OE*

*OsGRF1* has already been identified as the target of miR396 in plants [14, 15, 40]. A gain-of-function mutant overexpressing a microRNA-resistant-version of target has been used for elucidating the roles of a given microRNA (Axtell and [1]). *OsGRF1* was found to be highly expressed in shoot tips and young leaves (Fig. 1), indicating it likely plays role in regulating the growth of leaf and shoot. To avoid being targeted by OsmiR396, we got a miR396-resistant-version of *OsGRF1* by mutating five bases of *OsGRF1* mRNA in miR396-acted region without alteration of amino acid sequence according to the degeneracy of codons (Fig. 3a). Then we introduced miR396-resistant-version of *OsGRF1* (named as *mOsGRF1*) and wild-type *OsGRF1* into rice respectively, both were driven by the native promoter of *OsGRF1*. The transgenic plants were propagated and the homozygous lines were selected on *hygromycin* in T_2_ generation. Northern blot and qRT-PCR showed that the RNA abundance of miR396 was nearly at same level in 4-week-old seedlings of the non-transformed plants, *OsGRF1OE* and *mOsGRF1OE*, but the *OsGRF1* mRNA levels in *mOsGRF1OE* lines were significantly higher than that of the other two lines (Fig. 3b). The overall growth rates of the three genetic backgrounds are close to each other but an overgrowth of leaves was observed in *mOsGRF1OE* lines (Table 1). The first leaf of seedlings in both wild type and *OsGRF1OE* was an incomplete leaf of which shape likes a needle, whereas that of *mOsGRF1OE* lines had a tongue-like shape with a leafstalk (Fig. 3d). The sizes of other leaves of *mOsGRF1OE* lines were also bigger than that of the non-transformed plants and *OsGRF1OE* in the 3-week-old seedlings (Fig. 3c). Besides these, no other obvious difference of characteristics was observed in the three backgrounds. These results showed that *OsGRF1* plays roles in promoting the leaf growth. It is intriguing that *mOsGRF1OE* lines had no apparent difference in stem growth even though mRNA of *OsGRF1* also highly accumulated in the stem (Fig. 1). We speculated that overexpression of only one member of this family in shoot tips where all 12 members are highly expressed [6] might not be sufficient to produce an apparent stem elongation.

**Table 1:** Comparisons of the leaf phenotypes of the non-transformed plants, *OsGRF1OE* and *mOsGRF1OE* as well as *OsGIF1RNAi* lines^a^

	Length and width of the first leaf^b^(cm)	Length and width of the second leaf^b^(cm)	Length and width of the third leaf^b^(cm)	Length and width of the fourth leaf^b^(cm)
Non-transformed plants^c^	0.54 ± 0.06 (L) 0.11 ± 0.01 (W)	1.51 ± 0.19 (L) 0.21 ± 0.03 (W)	5.03 ± 0.40 (L) 0.51 ± 0.04 (W)	15.00 ± 0.88 (L) 0.81 ± 0.06 (W)
*OsGRF1OE*^c^	0.6 ± 0.05 (L) 0.13 ± 0.01 (W)	1.60 ± 0.15 (L) 0.23 ± 0.03 (W)	5.50 ± 0.5 (L) 0.56 ± 0.04 (W)	15.5 ± 0.77 (L) 0.86 ± 0.05 (W)
*mOsGRF1OE*^c^	0.91 ± 0.07 (L) * 0.31 ± 0.015 (W) *	3.11 ± 0.22 (L) * 0.40 ± 0.04 (W) *	8.41 ± 0.71 (L) * 0.70 ± 0.06 (W) *	19.02 ± 1.12 (L) * 1.1 ± 0.9 (W) *
*OsGIF1RNAi*^c^ *mOsGRF1OE* *×* *OsGIF1RNAi*^c^	0.33 ± 0.04 (L) * 0.07 ± 0.01 (W) * 0.37 ± 0.05 (L) * 0.08 ± 0.01 (W) *	0.85 ± 0.15 (L) * 0.16 ± 0.02 (W) * 0.91 ± 0.12 (L) * 0.17 ± 0.03 (W) *	3.8 ± 0.35 (L) * 0.41 ± 0.04 (W) * 4.1 ± 0.41 (L) * 0.48 ± 0.04 (W) *	13.20 ± 0.65 (L) * 0.72 ± 0.05 (W) * 13.90 ± 0.81 (L) * 0.81 ± 0.04 (W) *

^a^ Values are n ± SD

^b^ Statistical data are come from 3-week-old seedlings and the width presented to the widest section of leaves. L in brackets indicated the length and W in brackets indicated the width

^c^ Seven plants of each genetic background were analyzed

* Means Significant difference at *P* < 0.01 compared with the leaf data of non-transformed plants by Student’s t-test (*n* = 7; means ± SDs)

### Knockdown of *OsGRF1* by RNAi displays a phenotype of shrunken leaves

**Fig. 4:**
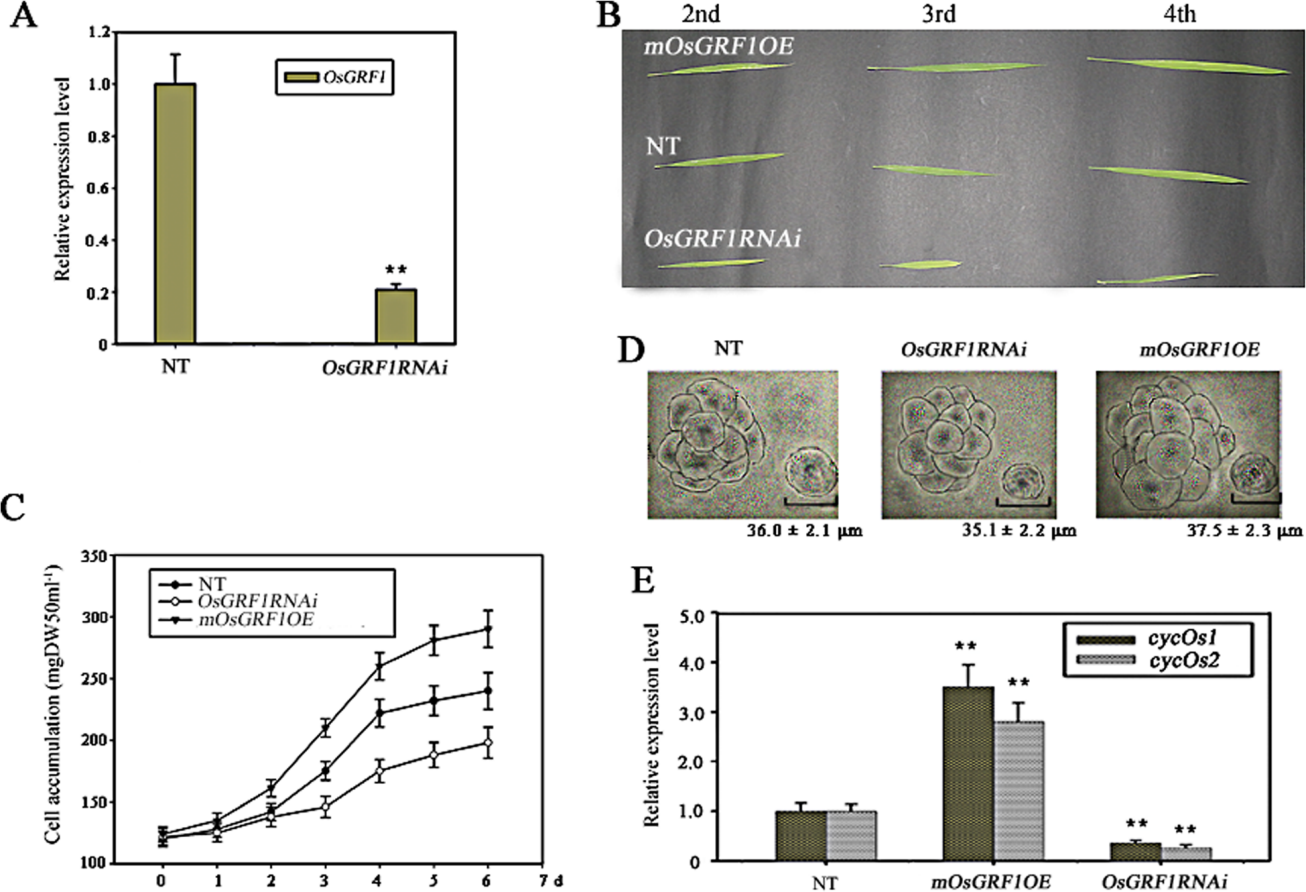
The phenotypes of transgenic lines with ectopic expression of *OsGRF1*. **a** The expression levels of *OsGRF1* in 2-week-old seedlings of the non-transformed plants (NT) and *OsGRF1RNAi*. Expression was analyzed by qRT-PCR. **, Significant difference at *P* < 0.01 compared with expression in the non-transformed plants (NT) by Student’s t-test (n = 3; means ± SDs). **b** The phenotype of the complete leaf from second to fourth position in the 3-week-old seedlings of the three backgrounds of *OsGRF1* (WT, *mOsGRF1OE*, *OsGRF1RNAi*). Bar = 10 cm. **c** The growth curve of the biomass of the suspension-cultured cells in the three backgrounds of *OsGIF1*. The suspension-cultured cells were harvested, dried, and weighed at given time. DW: dry weights. The data were the means of three biological repetition ± SE. **d** The morphology and size of the cells in suspension-cultured system originated from leaf *calli* in the three backgrounds of *OsGRF1*. Bar = 50 μm. **e** The expression levels of *cycOs1* and *cycOs2* in the leaves of three backgrounds of *OsGRF1*. Expression was measured by qRT-PCR. **, Significant difference at *P* < 0.01 compared with expression in the non-transformed plants (NT) by Student’s t-test (n = 3; means ± SDs)

To further investigate the roles of *OsGRF1* in rice development and growth, we knocked down *OsGRF1* by RNAi (RNA interference) technology. We chose a specific sequence corresponding to 3′ region of *OsGRF1* as object to construct RNAi vector and brought it into rice by *Agrobacterium*-mediated transformation*.* As shown in Fig. 4a, the expression of *OsGRF1* measured by qRT-PCR was significantly lower in the *OsGRF1RNAi* lines. The most prominent phenotype of knockdown of *OsGRF1* was that the transgenic lines exhibited smaller leaves (Fig. 4b). The differences between leaf sizes of the three genetic backgrounds (NT, *mOsGRF1OE*, *OsGRF1RNAi*) were more remarkable in the position closer to the base (Fig. 4b, Table 1). To investigate whether the difference in leaf growth was caused by cell proliferation or cell elongation, suspension-cultured cells stemmed from leaf *calli* of the three genetic backgrounds of *OsGRF1* were made. Plants suspension systems usually are made up of numerous lumps in which dozens cells clump together, and very few dissociated cells can be observed in suspension-cultured system. After 6 days of being cultured, the biomass increment of suspension-cultured cells of *OsGRF1RNAi* lines was significantly lower than that of the non-transformed plants, while that of *mOsGRF1OE* lines was remarkably higher (Fig. 4c). Additionally, there were no significant differences in the size of dissociated cells between the three genetic backgrounds (Fig. 4d). These results showed that the bigger differences of the leaf size between the three genetic lines are like caused by the activities of cell diversion rather than cell elongation. Some cell-cycle-related genes such as *cyclin Oryza sativa1* (*cycOs1*), *cyclin Oryza sativa2* (*cycOs2*) have been believed to be GA-induced [38, 39] even though it remained unknown whether these cell-cycle-related genes were related with *OsGRF1*. We measured the expressions of *cycOs1* and *cycOs2* in the leaves of 3-week-old seedlings of the three backgrounds of *OsGRF1.* As shown in Fig. 4e, the expressions of *cycOs1* and *cycOs2* are upregulated in lines of *mOsGRF1OE* and downregulated in lines of *OsGRF1RNAi*. These results fully demonstrated the activities of cell division could be affected by *OsGRF1* in rice leaf.

### Knockdown of *OsGIF1* exhibits pleiotropic growth defects including shrunken leaves while overexpression shows no variation

**Fig. 5:**
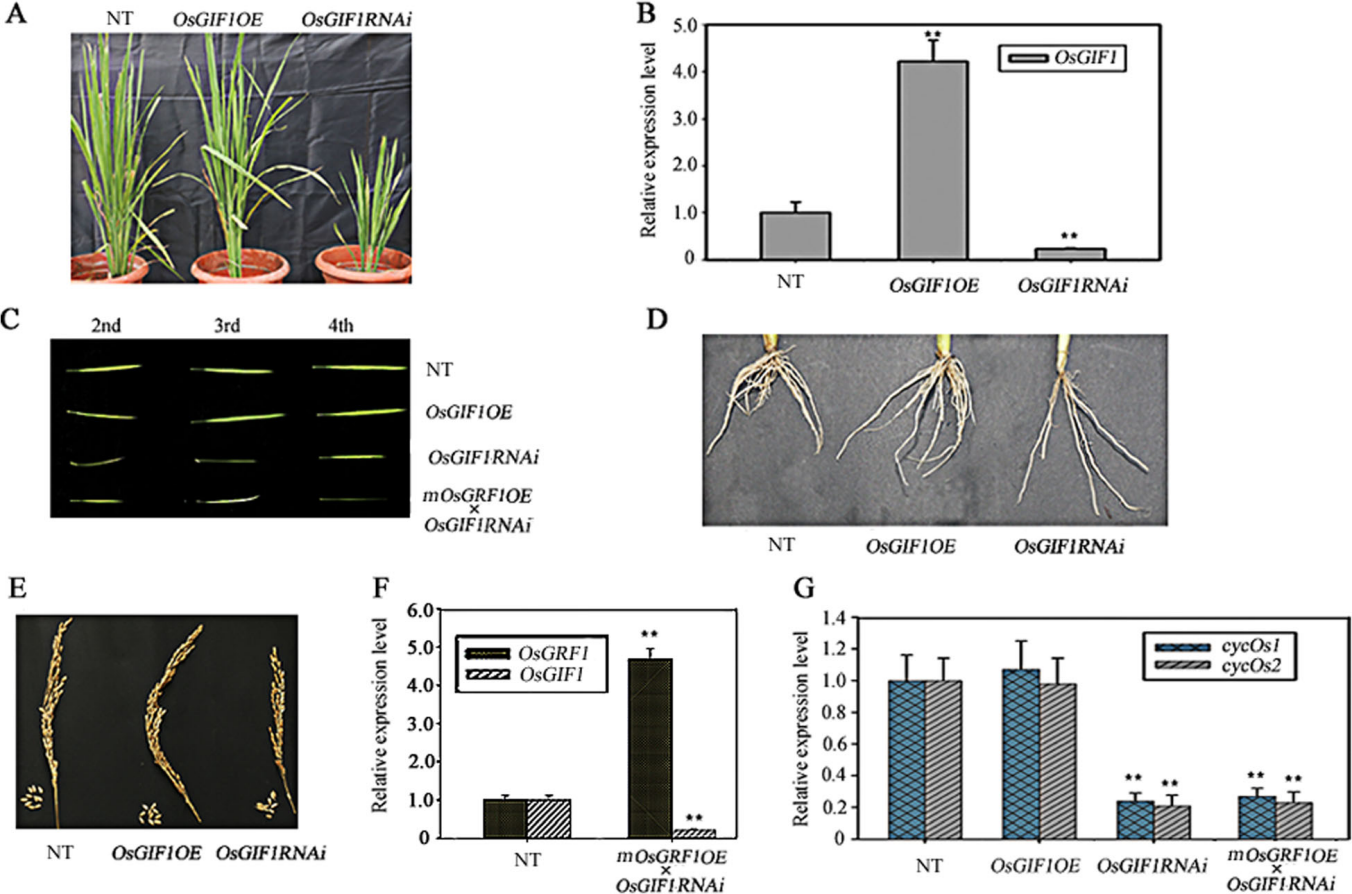
The phenotypes of the transgenic lines with ectopic expression of *OsGIF1*. **a** The phenotypes of the three genetic backgrounds of *OsGIF1* (NT, *OsGIF1OE*, *OsGIF1RNAi*). **b** The expression level of *OsGIF1* measured by qRT-PCR in the three genetic backgrounds of *OsGIF1*. **, Significant difference at *P* < 0.01 compared with expression in the non-transformed plants (NT) by Student’s t-test (n = 3; means ± SDs). **c** The phenotypes of the complete leaf from second to fourth position in the 3-week-old seedlings of the three backgrounds of *OsGIF1* as well as crossed line: *OsGIF1RNAi* × *mOsGRF1OE*. **d** The morphologic features of roots of the three backgrounds of *OsGIF1*. **e** The morphologic features of seeds and spikes of the three backgrounds of *OsGIF1*. **f** The expression level of *OsFRF1* and *OsGIF1* measured by qRT-PCR in 2-week-old seedlings of the non-transformed plants and crossed lines of *mOsGIF1RNAi* × *mOsGRF1OE*. **, Significant difference at *P* < 0.01 compared with expression in the non-transformed plants (NT) by Student’s t-test (n = 3; means ± SDs). **g** The expression level of *cycOs1* and *cycOs2* measured by qRT-PCR in leaves of the three backgrounds of *OsGIF1* as well as crossed line, *mOsGIF1RNAi* × *mOsGRF1OE*. **, Significant difference at *P* < 0.01 compared with expression in the non-transformed plants (NT) by Student’s t-test (n = 3; means ± SDs)

To investigate the functions of *OsGIF1* in rice, we made transgenic lines with overexpression or knockdown (RNAi by specific sequence) of *OsGIF1*. Then *OsGIF1* expression levels were measured by qRT-PCR in the three backgrounds (NT, *OsGIF1OE*, *OsGIF1RNAi*) (Fig. 5b). Expectedly, the expression of *OsGIF1* was significantly higher in *OsGIF1OE* lines while much lower in *OsGIF1RNAi* lines (Fig. 5b). Then the growth traits were carefully investigated during the whole lifetime. The plants overexpressing *OsGIF1* did not display any new phenotype all the time in their life cycle (Fig. 5a). Their stem length, tiller number, leaf size, and thousand-grain weight, are identical to the non-transformed plants (Fig. 5a). However, the knockdown lines of *OsGIF1* displayed multiple defects in their lifecycle, such as shorter stems, withered seeds, slender roots with reduced number, and shrunk leaves (Fig. 5c to e; Table 1). We had previously shown that the expressions of *cycOs1* and *cycOs2* could be affected by *OsGRF1* in the leaves (Fig. 4e), but we did not know whether this impact needs the partner of *OsGIF1*. To determine this uncertainty, the expressions of *cycOs1*and *cycOs2* were also measured by qRT-PCR in the leaves of the three genetic backgrounds of *OsGIF1*. As shown in Fig. 5g, the expressional levels of *cycOs1*and *cycOs2* were significantly lower in *OsGIF1RNAi* lines while did not change apparently in the *OsGIF1OE* lines (Fig. 5g). These results indicated the activity of cell division was inhibited in the leaves of the knockdown lines. For the reason why the expressions of *cycOs1*and *cycOs2* were not changed in the lines with overexpression of *OsGIF1*, we speculated that the expressions of *cycOs1*and *cycOs2* are under control of OsGIF1-OsGRF1 duo in which the expression of *OsGIF1* is already in an excessive state in the non-transformed plants (Fig. 1b and c). This assumption was further supported by the observation that the size of leaves still exhibited as shrunk in the crossed lines of *mOsGRF1OE* and *OsGIF1RNAi* (Fig. 5c), because only overexpression of *mOsGRF1* but lack the partner of *OsGIF1* is not enough to promote leaf growth. This observation was also supported by molecular evidence that the expression of *cycOs1*and *cycOs2* were still significantly lower in the leaves of hybrid lines of *mOsGRF1OE* and *OsGIF1RNAi* (Fig. 5g), in which the expression of *OsGRF1* is higher while that of *OsGIF1* is lower (Fig. 5f).

## Discussion

The roles of GRF-GIF duo have been revealed to be involved in many aspects of plant development and growth [15, 31]. However, compared with GIF family which usually comprises very few members, the GRF family is much bigger. So, the diverse functions of *GRFs* involving many aspects of plants development may reflect the combinations of the specific individual role of different family members. The roles of single member of *GIFs* seemed to be more versatile due to very fewer members in this family. *GIF* family found in most eukaryotic species is more conserved than *GRF* family [17], indicating they may have other roles beyond combination with *GRFs*. The observation that *Arabidopsis gif1/2/3* triple mutant displayed severe defects in the growth and development [22] further supports this assumption. So far, most studies focused on revealing the roles of individual member of the two families but not on the distinct roles of the different combinations [7, 24, 26, 30, 45, 48, 52]. Currently, the precise correlation between the individual members of the two families remains largely unclear.

Here we elaborately compared the expression patterns of *OsGRF1* and *OsGIF1* and profoundly analyzed the overlap of the phenotypes of transgenic plants with ectopic expression of *OsGRF1* and *OsGIF1*. From our results we can draw the key points as the following.

1. The expression of *OsGIF1* is in a constitutive manner with much higher levels while the expression levels of *OsGRF1* are in a tissue-specific preference with relative lower levels overall (Fig. 1). The reasons for higher expression level of *OsGIF1* are likely caused by two aspects: the lower copies of *OsGIFs* (only two in rice) in this family and the assumptions that GIFs also probably interact with other transcription factor, in addition to GRFs, based on some ChIP assays [45, 52];
2. The specific roles of *OsGRF1* may only be involved in regulating leaf growth while the roles of *OsGIF* may be involved in various aspects of plants growth. To explore the roles of *OsGRF1*, which prefers to express in tips of both stem and leaf, we use its native promoter rather than a constitutive promoter because we did not intend to sabotage its inherent expression way. The specific role of *OsGRF1* in regulating leaf growth was manifested due to avoid being targeted by miR396 (Fig. 3c and d). Additionally the observation that shrunk size of leaves also emerged in the lines of knockdown of *OsGRF1* by RNAi, further suggesting its role in regulating leaf growth (Fig. 4b). However the phenotype of knockdown of *OsGIF1* displayed multiple defects including shrunk leaves, indicating it may have multiple roles in plant growth (Fig. 5c to e). For reasons why overexpression of *OsGIF1* had no new phenotype, we speculated this would be caused by the fact that the expression of *OsGIF1* is already in an excessive manner in various tissues (Fig. 1b and c);
3. The expression of *OsGRF1* can be affected by various stresses and some kinds of hormones while that of *OsGIF1* is unaffected. The expressions of cell-cycle-related genes such as *cycOs1*and *cycOs2* in rice leaf are under control of OsGIF1-OsGRF1 duo. Even *OsGRF1* and cell-cycle-related genes such as *cycOs1* and *cyclin* were believed to be induced by GA [6, 38, 39, 43, 44], but it remained unknown whether there is a link between OsGRF1 and cell-cycle-related genes in GA response. Here we suggest that *cycOs1*and *cycOs2* are in the downstream of *OsGRF1* in response to GA because higher level of *OsGRF1* promoted the expression of *cycOs1*and *cycOs2* while lower level of *OsGRF1* inhibited them (Fig. 4e). Even *OsGIF1* did not respond to GA (Fig. 2a), the fact that *OsGRF1* can interact with *OsGRF1* and *OsGRF1RNAi* also presented a phenotype of shrunken leaves (Fig. 5c), indicating that the expression of *cycOs1*and *cycOs2* may need OsGIF1-OsGRF1 duo.

In summary, here we probed into the distinct role of a combination between the given members of *OsGRFs* and *OsGIFs*, and found their specific function in regulating leaf growth. The future studies may probably focus more on revealing the distinct roles of different combinations of *OsGRFs* and *OsGIFs.*

## Conclusions

**Fig. 6:**
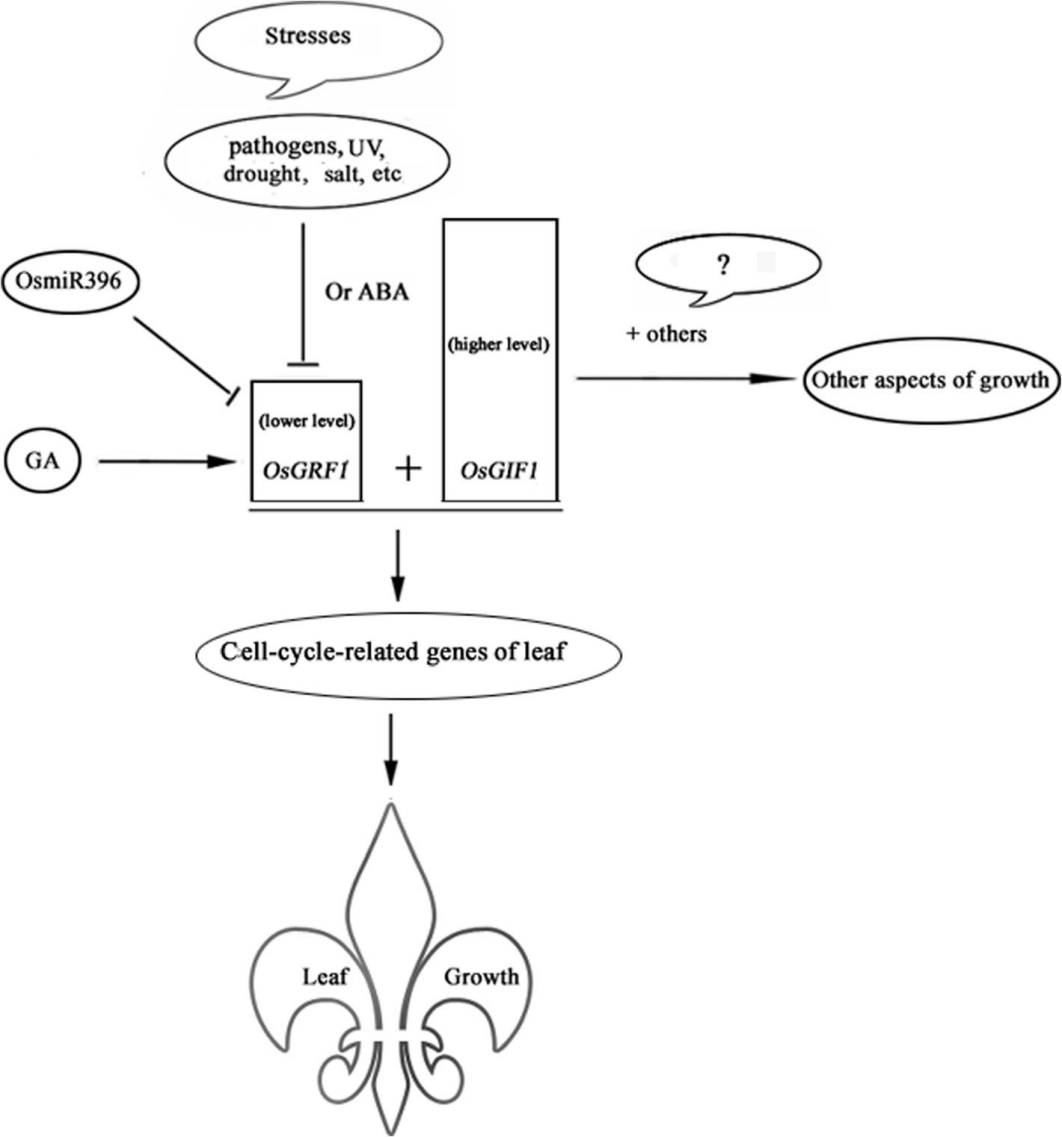
Model for the functions of *OsGRF1* and *OsGIF1* in regulating growth. ABA, OsmiR396, and various stresses such as pathogens, ultraviolet (UV), drought, salt, etc., can downregulate the expression of *OsGRF1*, which is usually in a lower level, while gibberellin (GA) upregulates it. The interaction between *OsGRF1* and *OsGIF1* is necessary in promoting leaf growth via promoting the expression of cell-cycle-related genes. OsGIF1, which expresses in a higher level, may also work with other factor(s) to regulate other aspects of growth

Based on the above results, we propose a working model here to interpret how *OsGRF1* and *OsGIF1* work together in regulating growth (Fig. 6). We suggest that the expression of *OsGRF1* is suppressed by various stresses, ABA, and miR396 while promoted by GA. The interaction between *OsGRF1* and *OsGIF1* is necessary in specifically promoting leaf growth by promoting the expression of cell-cycle-related genes. However, *OsGIF1*, which expresses in a higher level, may also work with other factor(s) in regulating other aspects of growth.

## Methods

### Plant materials and growth conditions

Rice cultivar (*Oryza sativa ssp. japonica*) was used as the control plants and serves the genetic background for all transgenic plants. All seeds involved in this study were taken from Key Laboratory of Plant Functional Genomics of the Ministry of Education, Yangzhou University, China. Y.L. undertook the formal identification of the plant materials used in his study. The voucher specimen of this material has not been deposited in a publicly available herbarium yet. The conditions for normal growth of the control plants and transgenic plants were performed as described by Lu et al. [28].

### Stress and hormone treatments

Salt stress, UV light stress, pathogen (*Magnaporthe grisea*) stress, drought stress, and abscisic acid (ABA) treatment, were performed as described previously [28]. For gibberellin (GA) treatment, the whole 2-week-old seedlings were incubated into N6 liquid solution containing 50 μM GA_3_ and 0.02% Tween 20. Then total RNA was extracted from the above seedlings at the point of designated time for genes analysis.

### Observation of cells and creation of suspension-cultured cells

For creation of suspension cells, the rice *calli* derived from sterilized leaves of the non-transformed plants, *OsGRF1OE*, and *mOsGRF1OE* lines were grown on N6 culture medium (solid). Four weeks later, 1 g of the fresh calli from different lines was incubated into 500 mL AA medium [41] and biomass was measured at given intervals. The suspension cells were observed and recorded under microscope.

### Quantitative RT-PCR

For quantitative RT-PCR analysis of *OsGRF1* (Gene ID: Os02g0776900), *OsGIF1* (Gene ID: Os03g52320), *cycOs1* (Gene ID: Os04g0563700) and *cycOs2* (Gene ID: Os06g0726800), 2 μg of total RNA was reversely transcribed in a total volume of 20 μL with 0.5 mg oligo (dT)15, 0.75 mM dNTPs, 10 mM DTT, and 100 U SuperScript II RNase H2 reverse transcriptase (Invitrogen). The reaction volume for PCR was 20 μL with 1 μL of the RT reactions [28]. The primers for quantitative RT-PCR are listed as the following: OsGRF1, FW: 5′-TGATCTTTCAAAAGAGGACGACG-3′, RV: 5′-TGGTGGTGATCGGGAGGTCGTT-3′; OsGIF1, FW: 5′-GCAGCAGCAGCAGGCGGCGGC-3′, RV: 5′-TGCCCTTGAGGTACTCCCCGT-3′; cycOs1: FW: 5′-GTGTTCTAGGATGATGGTAGA-3′, RV: 5′-GTTGTAACCTCCTGCTCCTGACT-3′, cycOs2: FW: 5′-CATGAGAAGGTTCCTCAAGGCT-3′, RV: 5′-TGGTGCACTGAGCAGTGTAGA-3′; 30 cycles for PCR was performed and the expression levels of the samples were normalized by *OsUbiquitin* gene (Forward: 5′-AACCAGCTGAGGCCCAAGA-3′, Reverse: 5′-AACCAGTCCATGAACCCGG-3′). Experiments were performed with three biological replicates, of which each was performed in three technical replicates.

### Northern-blot analysis

Total RNA was extracted from different tissues by using TRIzol reagent (Invitrogen). The DNA oligonucleotides of 5′-CAGTTCAAGAAAGCTGTGGAA-3′ served as probe for miR396, 5′-ATTTCTCGATTTGTGCGTGTC-3′ for U6; The two probes were labeled with γ-^32^P-ATP at 5′ terminal. For mRNA gel-blot analysis, The gene-specific probes for *OsGRF1*and *OsGIF1* were prepared by PCR amplification of genomic DNA that corresponded to the 3′ sequences of cDNA of the two genes and labeled with radioactive ^32^P (α-^32^P-dCTP). The two probes were designated to contain same content of radioactive ^32^P by designed primers (OsGRF1, FW: 5′-TGATCTTTCAAAAGAGGACGACG-3′; RV: 5′-TGGTGGTGATCGGGAGGTCGTT-3′; OsGIF1, FW: 5′-GCAGCAGCAGCAGGCGGCGGC-3′; RV: 5′-TGCCCTTGAGGTACTCCCCGT-3′). The process was performed as described previously [28].

### Construction of expression vector and generation of transgenic rice lines

The wild-type *OsGRF1* was firstly cloned by RT-PCR with primer as the following: FW: AAGGATCCCAGAGATGATGATGATGAGCGGTCG; RV: GCGAGCTCAGATTAATCATGCGGGAGGTGGTG. Then the miR396-resistant version of *GRF1* (*mGFF1*) was obtained by using mutagenic primers (FW: 5′- AAGCACATGCACCGTGGCAAGAACCGATCTAGAAAACCGGTGGAGATGTCCTTGGCCAC-3′; RV: 5′-CAAGGACATCTCCACCGGTTTTCTAGATCGGTTCTTGCCACGGTGCATGTGCTTCTCGCAGTAC-3′). During process of mutation, the first-round PCR products were purified and used as a template for the second amplification. The resulting product was then digested and cloned into pUC18 and the positive clone was verified by sequencing. Finally, both mutated and wild-type *OsGRF1* were brought into pCAMBIA1301 in which the original *Ubi1* promoter was replaced by the promoter of *OsGRF1*. The full length of *OsGIF1* was cloned by the RT-PCR with primers as the following: FW: 5′-ATGCAGCAGCAACACCTGATGC-3′; RV: 5′-CTAGCTGCCTTCCTCCTCGGT-3′. The OsGIF1 was then constructed into pCAMBIA1301 under Ubi1 promoter for overexpression. For RNAi (RNA interference) of both OsGRF1 and OsGIF1, the specific regions (probe region for northern blot) of the two genes were used for silencing the targets and were brought into pCAMBIA1301 forward and backward respectively, separated by an intron. All the constructed expression vectors were introduced into rice *calli* through *Agrobacterium tumefaciens* (EHA105) mediated methods [12].

### Western blot

For western blot of OsGRF1 and OsGIF1, total protein from the 2-week-old seedlings of the non-transformed plants was extracted by SDS sample buffer and boiled for 10 min. Then the extracted proteins were separated by SDS-PAGE and immunoblotted with antibody of anti-OsGRF1 and anti-OsGIF1 at 1:1000 dilution.

To prepare the antibodies of OsGRF1 and OsGIF1, 6 × His-OsGIF1 and 6 × His-OsGRF1 constructed into pET28 vector were expressed and used as antigens to produce monoclonal antibodies in rabbits (Purchased from Junhui Biotech, Co, China). During the process of making monoclonal antibodies, the rabbits were immunized four times at least and the purities of the extracted antibodies should be kept greater than 90%. Finally, the values of enzyme linked immunosorbent assay (ELISA) should be greater than 1:128000.

## Data Availability

The datasets generated and analyzed during the current study are available from the corresponding author on reasonable request. Sequence data from this article can be found from the database (http://www.ricedata.cn/gene/) under the following gene ID’s/accession numbers: *OsGRF1* (Os02g0776900), *OsGIF1* (Os03g52320), *cycOs1* (Os04g0563700) and *cycOs2* (Os06g0726800).

## Abbreviations

ABA: Abscisic acid
GRFs: Growth-regulating factors
GIFs: GRF-Interacting Factors
NLS: Nuclear localization signal
qRT-PCR: Quantitative Reverse-Transcription Polymerase-Chain-Reaction
SAM: Shoot Apical Meristem
RNAi: RNA interference
